# Percutaneous treatment of native aortic coarctation in adults

**DOI:** 10.1007/s12471-012-0290-x

**Published:** 2012-06-01

**Authors:** P. Luijendijk, S. M. Boekholdt, B. J. M. Mulder, R. J. de Winter

**Affiliations:** 1 Department of Cardiology, Academic Medical Centre, B2-216 Meibergdreef 9, 1105 AZ Amsterdam, the Netherlands; 2 Department of Cardiology, Academic Medical Center, Room F3-240 Meibergdreef 9, 1105 AZ Amsterdam, the Netherlands; 3 Cardiology Department, Academic Medical Center, B2-240 Meibergdreef 9, 1105 AZ Amsterdam, the Netherlands; 4 Department of Cardiology, Academic Medical Center, B2-137 Meibergdreef 9, 1105 AZ Amsterdam, the Netherlands

Dear Editor,

With great interest we read the response of Walhout et al. to our case description of two native coarctation patients in which percutaneous angioplasty with stenting was performed [1]. Walhout et al. describe that the occurrence of recoarctation is limited in adult patients with native coarctation. They suggest that evidence for the additive value of stenting for the prevention of recoarctation is limited. Additionally they report that the occurrence of aneurysm formation is similar after balloon angioplasty with or without stenting.

The risk of aneurysm formation after balloon angioplasty reported by Walhout et al. and Fawzy et al. is indeed lower than reported in previous studies [2, 3]. However, this might be related to the fact that only patients with a discrete coarctation or ‘shelf-like’ lesion were included in these series, as previously hypothesised by Tanous et al. [4] Walhout et al. report that stenting does not prevent aneurysm formation. However, previous studies have shown that the risk for aortic aneurysm formation is 51 % after surgical repair, 17 % after balloon angioplasty and less than 10 % after coarctation with stenting [5, 6]. The long-term effect of covered stents in preventing aneurysm formation needs to be evaluated in larger series.

With regard to the risk of recoarctation, Rodes-Cabau et al. have reported in a series of 80 consecutive children (age 12 ± 10 years) treated with either angioplasty or surgical repair that 18 % of all patients required reintervention for recoarctation [7]. However, studies performed in adults suggest that the risk of recoarctation is still higher after treatment with balloon angioplasty without stenting compared with treatment with additional stenting [6, 8]. These findings suggest that in adults with native aortic coarctation, angioplasty with stenting reduces the risk of recoarctation and might prevent aneurysm formation as compared with balloon angioplasty alone. According to the current guidelines, stenting has become the treatment of first choice in adults with native and recurrent coarctation, although the use of covered or non-covered stents is still a matter of debate [9]. In our opinion, stenting is therefore still advocated in adult native coarctation patients, as it might prevent vessel elastic recoil and reduce the need for reinterventions during follow-up.

## References

[1] Luijendijk P, Boekholdt SM, Blom NA (2011). Percutaneous treatment of native aortic coarctation in adults. Neth Heart J.

[2] Fawzy ME, Awad M, Hassan W (2004). Long-term outcome (up to 15 years) of balloon angioplasty of discrete native coarctation of the aorta in adolescents and adults. J Am Coll Cardiol.

[3] Walhout RJ, Suttorp MJ, Mackaij GJ (2009). Long-term outcome after balloon angioplasty of coarctation of the aorta in adolescents and adults: Is aneurysm formation an issue?. Catheter Cardiovasc Interv.

[4] Tanous D, Collins N, Dehghani P (2010). Covered stents in the management of coarctation of the aorta in the adult: initial results and 1-year angiographic and hemodynamic follow-up. Int J Cardiol.

[5] Forbes TJ, Garekar S, Amin Z (2007). Procedural results and acute complications in stenting native and recurrent coarctation of the aorta in patients over 4 years of age: a multi-institutional study. Catheter Cardiovasc Interv.

[6] Zabal C, Attie F, Rosas M (2003). The adult patient with native coarctation of the aorta: balloon angioplasty or primary stenting?. Heart.

[7] Rodes-Cabau J, Miro J, Dancea A (2007). Comparison of surgical and transcatheter treatment for native coarctation of the aorta in patients > or =1 year old. The Quebec Native Coarctation of the Aorta study. Am Heart J.

[8] Pedra CA, Fontes VF, Esteves CA (2005). Stenting vs. balloon angioplasty for discrete unoperated coarctation of the aorta in adolescents and adults. Catheter Cardiovasc Interv.

[9] Baumgartner H, Bonhoeffer P, De Groot NM (2010). ESC Guidelines for the management of grown-up congenital heart disease (new version 2010). Eur Heart J.

